# Dorsal posterior cingulate cortex encodes the informational value of feedback in human–computer interaction

**DOI:** 10.1038/s41598-020-68300-y

**Published:** 2020-08-03

**Authors:** Susann Wolff, Christin Kohrs, Nicole Angenstein, André Brechmann

**Affiliations:** Combinatorial NeuroImaging Core Facility, Leibniz Institute for Neurobiology, 39118 Magdeburg, Germany

**Keywords:** Neuroscience, Psychology

## Abstract

In communication between humans as well as in human–computer interaction, feedback is ubiquitous. It is essential for keeping up the dialogue between interaction partners, evaluating the adequacy of an action, or improving task performance. While the neuroscientific view on feedback has largely focused on its function as reward, more general definitions also emphasise its function as information about aspects of one’s task performance. Using fMRI in a computer-controlled auditory categorisation task, we studied the neural correlates of the informational value of computer-given feedback independent of reward. Feedback about the correctness of a decision, compared with feedback only indicating the registration of a decision, increases activation of the dorsal posterior cingulate cortex, supporting this region’s role in adapting to behaviourally relevant information. Both conditions elicit equally strong activation of the dorsal striatum which does not support an interpretation of feedback information as a type of reward. Instead, we suggest that it reflects a more fundamental aspect of human interaction behaviour, namely the establishment of a state that enables us to continue with the next step of the interaction.

## Introduction

Feedback is essential in human dialogues as well as for a successful communication in human–computer interactions (HCI)^1^. It is of particular importance in situations where humans are trying to improve their performance in a given task, which is the case in typical school scenarios as well as when someone is trying to excel in a computer game or interacting with an intelligent tutoring system. Even though a lot is known about the effects of feedback in such contexts from decades of behavioural research^2–6^, the underlying mechanisms of successful feedback application are vastly unexplained^5^.

More recently, neuroimaging studies have contributed to the understanding of these mechanisms by investigating how different functions of computer feedback are represented in the brain. This includes addressing the neuronal effects of feedback timing and reliability. While delayed and omitted computer feedback elicit behavioural and psychophysiological responses that reflect irritation and frustration of the user^7,8^, fMRI studies have shown that this is accompanied by a strong activation of a brain network for attention and action control^9,10^. Despite the fact that the feedback employed in these studies only registered the participants’ button press, differential activation for immediate, delayed, and omitted feedback was also found in brain areas typically associated with reward processing, e.g. the dorsal striatum^9^ (see also^11^ for a comparable study that used no feedback and non-contingent feedback as control conditions). Moreover, the activation differences resemble findings on temporal prediction error coding in monkeys^12^ and humans^13^ that, however, explicitly used rewarding feedback stimuli.

At first glance, these results may therefore appear to be in line with traditional reinforcement-based theories^14,15^ that inextricably linked feedback with reward (see^16^, for a historical perspective). Following this line of thinking, the vast majority of studies on the neural correlates of feedback processing have been designed as reinforcement experiments using reward and punishment, and as a consequence, any activation in dopaminergic brain regions has almost exclusively been attributed to mechanisms of reward processing^17,18^. In this context, the dorsal striatum has specifically been associated with the anticipation of rewarding feedback^19,20^ and the establishment of associations or contingencies between stimulus, response and reward^21–23^.

However, no reward in the classical sense (i.e. no primary homeostatic or reproductive reward, and no secondary reinforcer like money) was administered in the experiments described above^9,11^. By contrast, participants were informed that correct as well as incorrect decisions would be followed by the same neutral feedback (a check mark, a neutrally spoken “okay”). Consequently, we suggested that the involvement of the brain’s reward system may also reflect functions of feedback that are independent of reward-related processing. Similarly, Aron et al.^24^ (see also^25^) also found feedback-induced activation in dopamine-related areas that could not be fully explained by stimulus-reward associations, which led them to the conclusion that dopaminergic activation should be conceptualised more generally in terms of informationally salient events rather than specifically in terms of reward. Such a role of dopamine neurons being most active when critical information is available for learning has also been suggested by McGovern et al.^26^.

Thus, to fully understand the role of the reward system and other brain areas in the processing of (computer) feedback, we need to return to the question of the different functions of feedback. According to Mory^27^ (see also Kulhavy and Wager’s^16^ “feedback triad”), it is essential to separate the function of feedback as a motivator or reinforcer from its function as a unit of information (cf.^28^). Thus, while feedback may have a rewarding or motivating effect, its main function from an information-processing point of view is providing the user with an opportunity to correct errors, not to administer reward or punishment. In their seminal meta-analysis on the effects of feedback interventions on performance, Kluger and DeNisi^2^ accordingly define feedback as “actions taken by (an) external agent(s) to provide information regarding some aspect(s) of one’s task performance” (p. 255).

Crucially, however, feedback can convey more than one type of information. More precisely, neutral feedback (as employed in the studies described above^9,11^) might not be considered as classically informative in the sense of the definitions quoted above, since it does not relate to the correctness of the user’s responses, i.e. it is not evaluative. However, neutral feedback still provides the user with valuable information by indicating that the interaction has not been interrupted. Such registering feedback is also a prerequisite of functioning human-to-human dialogues: Imagine a conversation (e.g. on the telephone) where your interlocutor does not give any indication that he can hear your contributions. In HCI scenarios, where no information from other channels (such as nodding, mimics, gestures, or eye gaze) is available, the need for registering feedback is particularly pronounced. In this context, registering feedback fulfils the function of establishing common ground^29^, giving the user a sense of completion^30^, and informing him that he does not need to repeat his action^31^.

To summarise, besides a possible motivating or rewarding function, computer feedback is also able to fulfil an informative function on at least two levels: First, any type of correlated feedback allows the identification of a functioning dialogue by informing the user that his input has been registered. Second, feedback can additionally contain evaluative information about the user’s performance allowing the user to adapt his response behaviour if necessary. In how far the neural mechanisms underlying the processing of these different forms of feedback information differ from each other and to what extent they are comparable to the neural correlates of reward processing has, to the best of our knowledge, not been addressed so far.

In the current fMRI experiment, we therefore aimed to investigate the processing of the described two subtypes of informative feedback (registering vs. evaluative) by examining their differential effects on brain activation. To this avail, we employed an auditory categorisation task (cf.^8–11^) and compared the effects of registering computer feedback, i.e. feedback that merely informs the participant about the registration of his button press, with those of evaluative computer feedback, i.e. feedback that provides information about whether the button press was correct or incorrect. With regard to the brain regions involved in the differential processing of these two types of feedback information, we aimed at further disentangling the role of the dorsal striatum in human feedback processing. In this regard, the experiment served to contrast two competing approaches:

If activation of the brain’s reward system is interpreted as indicating the processing of reward and only reward, this would imply that activation of the dorsal striatum by registering feedback^9,11^ needs to be understood as an indicator that the informational value of registering feedback constitutes a form of reward in and of itself. In this case, we should expect an additional increase in activation by evaluative compared to registering feedback, since more information (registration information plus correctness of response information) should constitute a higher reward.

If, on the other hand, activation of the brain’s reward system by registering feedback specifically reflects the identification of a functioning dialogue, this type of activation should be observable for both registering feedback and evaluative feedback to an equal degree, because the continuation of a functional dialogue situation can be ascertained from both types of feedback. Localising the neural representation of the additional informational value of evaluative feedback as compared to registering feedback in this case requires to broaden the view beyond the brain’s reward system.

In addition to comparing the two feedback information types (registering vs. evaluative), we employed feedback of varying modalities. Depending on the requirements of the application, computer feedback can be presented in a multitude of modalities. A special role in this regard is played by spoken verbal feedback^32–34^ because of its predominance in human-to-human interactions which increases the demand for natural conversation with intelligent systems (e.g. chatbots, tutoring systems). In order to identify feedback effects that are independent of modality, we therefore provided registering as well as evaluative feedback in verbal form (speech), non-verbal auditory form (sinus tones), and visual form (symbols).

## Results

In the current experiment, participants were asked to categorise frequency-modulated tones according to their modulation direction by pressing one of two buttons for rising versus falling tones. In different blocks of the experiment, each participant received feedback that was (a) registering or evaluative, and (b) visual, auditory, or verbal. In the following, behavioural and neuroimaging results are summarised.

### Behavioural results

Table 1 shows the descriptive statistics (means and standard errors) for accuracy rates and reaction times for each of the six experimental conditions. Visual inspection of Q–Q plots, skewness (all $|\textit{S}| < 0.95$, all $\textit{p} > 0.05$), kurtosis (all $|\textit{K}| < 1.31$, all $\textit{p} > 0.05$), and Shapiro-Wilk tests (all $\textit{W} >0.91$, all $\textit{p} > 0.05$) showed no significant deviations from normal distribution. Neither of the repeated-measures ANOVAs revealed significant main effects or interaction effects of informational content and modality (all $\textit{F} < 3.79$, all $\textit{p} > 0.05$).

**Table 1:** Descriptive statistics (means, s.e.m.; $\textit{n} = 16)$ for participants’ accuracy rates and reaction times in trials with registering versus evaluative feedback, for each of the three feedback modalities (visual, auditory, verbal).

	Registering feedback	Evaluative feedback
**Accuracy (% correct)**		
Visual modality	89.5 (1.8)	89.1 (1.6)
Auditory modality	88.9 (2.0)	90.5 (1.5)
Verbal modality	90.1 (1.6)	90.9 (1.5)
**Reaction times (ms)**		
Visual modality	694 (22)	656 (22)
Auditory modality	695 (23)	673 (24)
Verbal modality	700 (21)	675 (23)

Standard errors of the mean are given in parentheses.

### fMRI results

**Figure 1:**
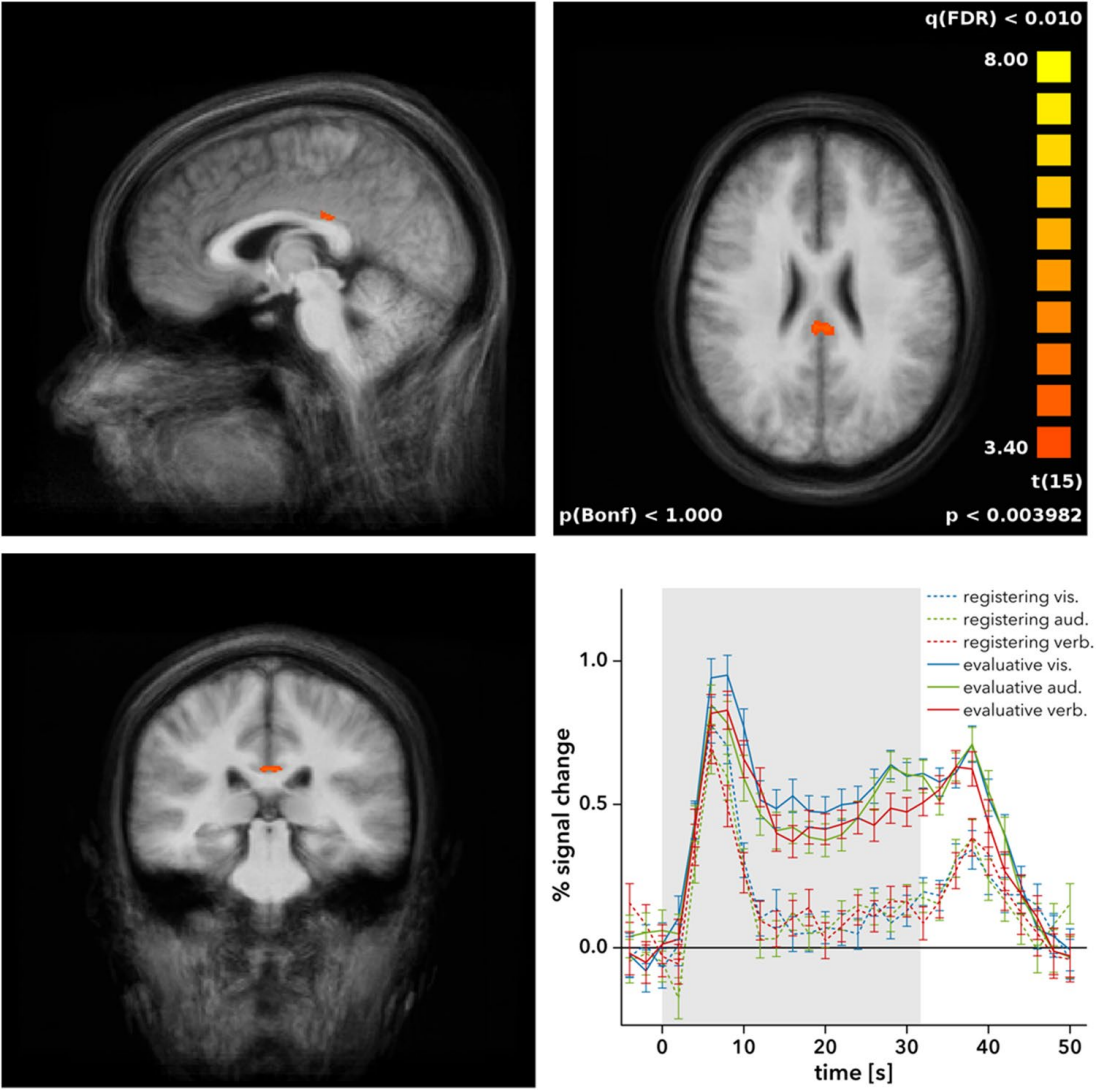
The posterior cingulate gyrus shows stronger activity during evaluative feedback compared to registering feedback. *Bottom right* The time course of the BOLD response in this region shows this difference in activation across all modalities. Error bars indicate s.e.m. *vis.* visual modality, *aud.* auditory modality, *verb.* verbal modality.

The fMRI results show a significantly stronger activity in the posterior cingulate gyrus (PCC; BA 23; Talairach coordinates: x = 0, y = − 28, z = 25; volume: $93\,\hbox {mm}^{3}$; mean *t* = 3.65; mean *p* = 0.003) in response to evaluative feedback compared to registering feedback regardless of modality (see Fig. 1). The evaluative feedback in all three modalities elicited a substantially higher BOLD response than the registering feedback, especially in the later phase of the blocks, where the BOLD response decreased almost back to baseline in the registering feedback conditions while it remained at a considerably higher plateau in the evaluative feedback conditions.

**Figure 2:**
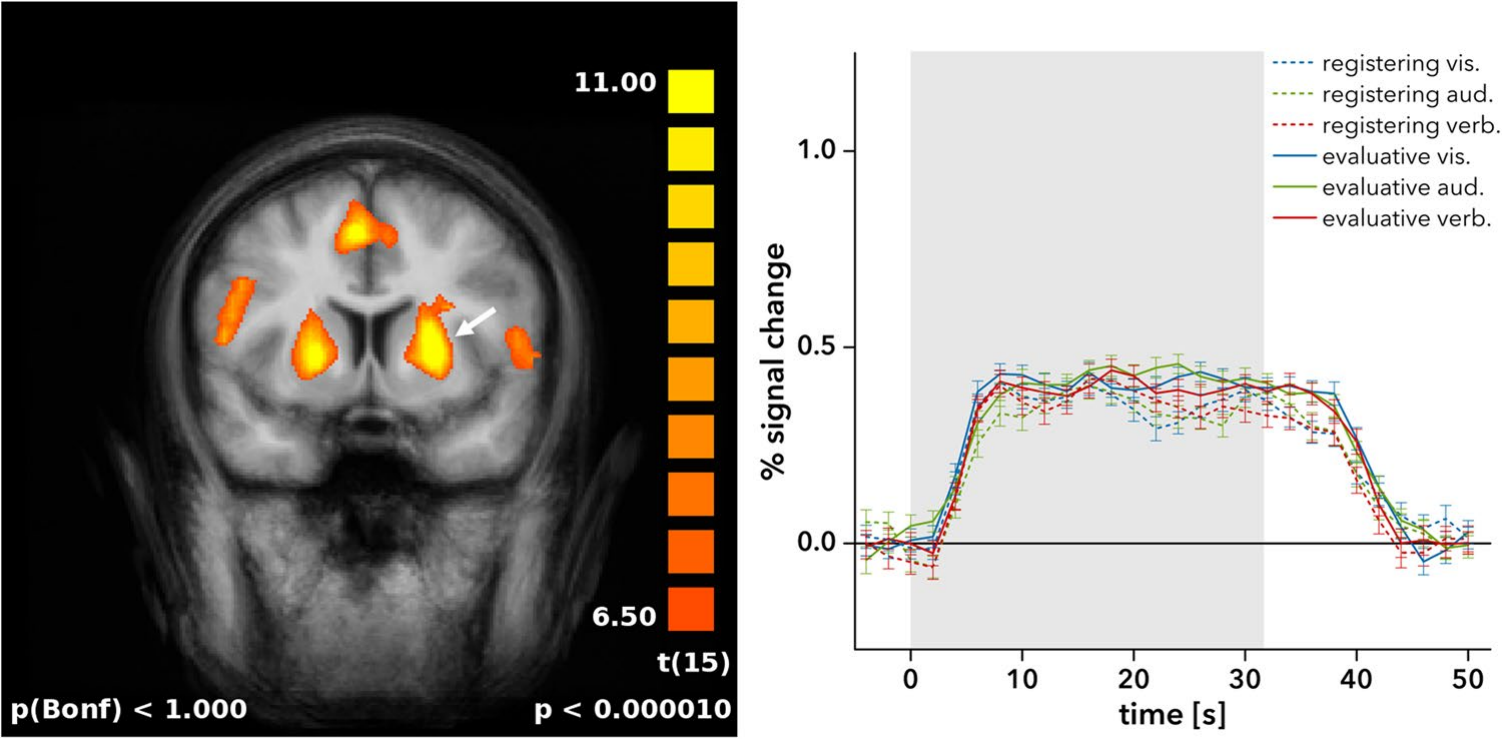
*Left* Increased activation ($\textit{t} > 6.5$) in the dorsal striatum for all feedback conditions versus fixation. *Right* The time course of the most significantly activated voxels (volume: $1000\,\hbox {mm}^{3}$) within the left dorsal striatum shows that all feedback types and modalities elicit equally strong BOLD responses. Error bars indicate s.e.m. *vis.* visual modality, *aud.* auditory modality, *verb.* verbal modality.

No other brain regions showed significant differences in neural activity in response to evaluative feedback compared to registering feedback. Specifically, no effects of informational content were apparent in the dorsal striatum or other areas associated with the human reward system. To substantiate this, we calculated a more sensitive general linear model on a region-of-interest level for all voxels covering the dorsal striatum area previously reported to be differentially activated in the same task by immediate, delayed and omitted registering feedback^9^ (Talairach coordinates: x = − 24, y = 9, z = 3; volume: $20\,\hbox {mm}^{3}$). This analysis confirmed the lack of differential feedback effects in this area (mean *t* = 0.94, mean *p* = 0.372), as did a corresponding analysis of the dorsal striatum area as identified by Behne et al.^11^ (Talairach coordinates: x = − 20, y = 6, z = − 2; volume: $760\,\hbox {mm}^{3}$; mean *t* = 0.78, mean *p* = 0.498). Illustrating this lack of differences, Fig. 2 shows the striatal activation in the current study for all feedback conditions versus rest/fixation (Talairach coordinates: x = − 22, y = 1, z = 9) and the equal BOLD signal increase that is elicited in this area throughout the entire task block by both evaluative and registering feedback.

## Discussion

The experiment described here aimed at distinguishing different types of feedback information in human computer interaction and their neural correlates. A particular focus was set on the role of dopamine-related brain areas, specifically the dorsal striatum, which in previous studies^9,11^ has demonstrated an increase in activation following correlated registering feedback that did not include any reward in the classical sense. To this avail, the neural responses to feedback with mere registering information were compared to the responses to feedback with additional evaluative information.

From a reward-centric perspective on feedback information in human–computer dialogues, i.e. building on the assumption that information itself has some reinforcing/rewarding properties and that more information would equal a higher reward, a stronger increase in activation in the dorsal striatum would have been expected in blocks with evaluative feedback compared to blocks with merely registering feedback. This, however, was not reflected in our results. Instead, we observed differential activation for evaluative versus registering feedback only in the posterior cingulate cortex (PCC), an area not typically referred to in studies on reward-related processing. In the dorsal striatum, on the other hand, we observed an equal increase in activation for both registering and evaluative feedback. This supports an understanding of the processing of feedback information as essentially independent from reward processing mechanisms.

In accordance with the interpretation given by Behne et al.^11^, the increase in activation that we observed in the dorsal striatum may well reflect a more basic process like the confirmation of a successful continuation of an ongoing interaction, something that is of fundamental importance especially in dialogues between humans and computers, where interruptions of communication commonly occur due to technical failure. Crucially, in the present experiment, both registering feedback and evaluative feedback were administered in a timely, correlated fashion, so that both types of feedback equally provided this essential information and, as a consequence, activated the dorsal striatum to an equal extent.

It is important to note that we do not mean to reinterpret previous findings of striatal activation in experimental situations where feedback in fact primarily fulfilled its function as a motivator/reinforcer^17,19–21,35^. Indeed, two previous studies showed that the addition of monetary rewards increased striatal activation as compared with purely evaluative feedback^36,37^. The vast majority of studies that reported striatal activation in response to feedback, however, inseparably coupled positive evaluative feedback with the prospect of monetary gain or other secondary reinforcers (e.g.^22,38–43^). When such reinforcement is not implied and feedback is stripped down to its informational content, however, we suggest that the remaining striatal activation does not reflect any reward-related processes but simply indicates the identification of a functioning dialogue, as was the case with both types of informative feedback in the current study.

An alternative interpretation of the equal striatal activation observed for all feedback conditions cannot be ruled out, since the accuracy in the employed categorisation task was very high: If information about successful performance (i.e., positive evaluative feedback) is perceived as rewarding/motivating to attain a performance goal^44^, one could argue that we observed striatal activation (a) in blocks with evaluative feedback since this consisted predominantly of positive feedback, and (b) also in blocks with registering feedback due to the high probability of the registered response being correct. While it is unclear to what degree participants were aware of this high probability of success, future experiments should manipulate task difficulty to address this possibility.

Beyond the discussion about the involvement of the reward system, we found that information about the adequacy of the user’s task performance, which was provided by evaluative but not by registering feedback, elicited an increase in activation in a region that has previously not been in the centre of attention in the area of feedback research. Based on its Talairach coordinates, this activation conforms to BA 23 and can therefore be considered as part of the posterior cingulate cortex (PCC). As of today, the functional role of the PCC is not yet clear^45–47^. First and foremost, the PCC is known as one of the most metabolically active brain regions at rest and has been set at the centre of the default mode network (DMN), which shows conjoint deactivation during the engagement in a wide range of attention-demanding, externally-oriented, goal-directed cognitive tasks^48–53^.

**Figure 3:**
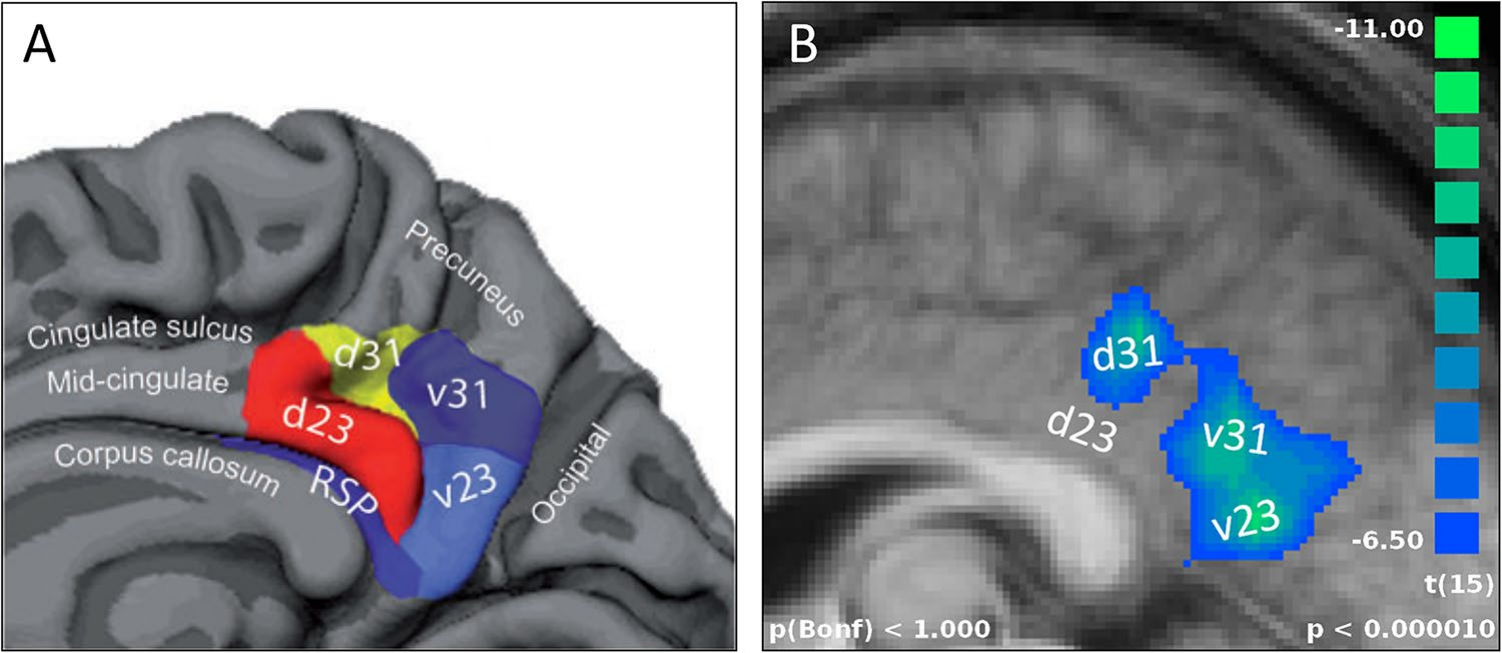
**A** Parcellation of PCC sub-regions based on Vogt^55^, adapted from Leech and Sharp^46^, with permission. **B** Areas of the PCC with DMN-typical deactivation ($\textit{t} < -6.5$) during blocks of task performance compared to blocks of rest, including sub-regions d31, v31, and v23.

In contrast to the task-induced deactivation typically associated with the PCC as part of the DMN, however, the cluster of PCC activation in the current study showed a pronounced positive deflection of the hemodynamic response function during the auditory categorisation task (see Fig. 1). To understand this ostensible contradiction, one needs to take into account that the DMN was originally defined based on low resolution PET data and early fMRI data using large smoothing kernels, which led to a treatment of the entire PCC as a uniform component of the DMN. In recent years, however, it has become clear that a more detailed parcellation of the PCC is required anatomically^54,55^ as well as functionally^46,47,56,57^. Building on Brodmann’s^58^ identification of cytoarchitecturally defined areas, Vogt and colleagues^54,55^ provided a novel anatomical parcellation scheme of the posterior cingulate cortex, dividing this brain area into a dorsal portion (dPCC, including the dorsal parts of BA 23 and BA 31) and a ventral portion (vPCC, including the ventral parts of BA 23 and BA 31). The resulting PCC sub-components (d23, d31, v23, v31) are illustrated in Fig. 3A.

Following Vogt and colleagues’ parcellation scheme^54,55^, the activation observed in the current experiment can be attributed to the dorsal part of BA 23 corresponding to PCC area d23 in Fig. 3A. To verify that the observation of a positive BOLD response of this area in blocks with evaluative feedback does not automatically imply a conjoint task-positive activation of the rest of the PCC/DMN, we performed an additional analysis contrasting the activation in blocks of task performance (with evaluative or registering feedback) with the activation in resting blocks. This did indeed reveal the DMN-typical deactivation during task performance (Fig. 3B). The affected PCC areas, however, were either located more superior or more posterior than the observed cluster of positive activation for evaluative versus registering feedback (cf. Fig. 1), thus conforming to areas d31 and v31/v23 of the PCC.

From this pattern of activation and deactivation in different sub-regions of the PCC it becomes clear that the cognitive processes underlying the observed activation in area d23 cannot be understood by reflecting on PCC function as an undifferentiated whole. On the basis of the anatomical organisation put forward by Vogt and colleagues^54,55^, Leech and Sharp^46^ reappraised the functional organisation of the PCC, specifically differentiating between the functions of the ventral and the dorsal portion of the PCC. In this framework, the so-called ABBA (Arousal, Balance and Breadth of Attention) model, activity of the ventral portion of the PCC is primarily associated with internally directed attention (see also^47,54,59^), which has also been advocated as one of the processes underlying DMN activation in general^52,60–63^. Activation of the dorsal portion of the PCC, on the other hand, is primarily associated with a broad external focus of attention, leading the authors to suggest an involvement in “detecting and responding to environmental events that may require a change in behavior”^46^ (p. 24).

Supporting evidence for the assumption that PCC sub-regions are involved in distinct cognitive processes and do not simply function as a unitary, undivided module of the DMN stems from functional connectivity analyses of fMRI data. For example, Leech et al.^56^ demonstrated that a strong functional connectivity of the PCC to the rest of the DMN is mainly exhibited by ventral portions of the PCC. The dorsal PCC, on the other hand, is functionally highly connected to frontoparietal networks of attention and executive control, suggesting a more prominent role of dPCC in the control of cognition^56^. Similarly, Fan et al.^57^ reported a higher connectivity of dPCC to the central-executive network (CEN) than to the DMN (as opposed to vPCC’s stronger connection to the DMN). Taken together, these observations strongly emphasise the need to consider more fine-grained specialisations of PCC subareas when discussing PCC function.

The present finding of an increased activity of the dorsal PCC (specifically area d23) during blocks with evaluative feedback is in accordance with Leech and Sharp’s^46^ conception of the dorsal PCC as involved in the behavioural adaptation to information from the environment. Based on its higher informational content, evaluative feedback conceivably possesses a higher relevance for behavioural adaptation than registering feedback. As a consequence, the observed increase of activity in area d23 may reflect the organism’s adjustment to the available information about the adequacy of the individual’s behaviour.

In this regard, it needs to be acknowledged that the current findings do not entirely support a functional dichotomisation between dPCC and vPCC as put forward by Leech and Sharp^46^, Bzdok et al.^47^, or Fan et al.^57^. Based on the differential activation and deactivation pattern in area d23 versus areas v23, d31, and v31 (see Fig. 3B), the current findings suggest an even closer correlation between the functional specialisation of the PCC and its anatomical parcellation as put forward by Vogt^55^, and thus a separate consideration of all four sub-regions of the PCC (cf. Fig. 3A). More precisely, such a more intricate differentiation could address the following mismatch between Leech and Sharp’s^46^ conceptualisation of the ABBA model and our data: While Leech and Sharp^46^ indeed predict an increase in neural activity in the dPCC for a broad external focus of attention, they associate a narrow external focus with deactivation of both vPCC and dPCC. The task employed in the current study, however, must primarily be considered as requiring such a narrow external focus of attention (attending task-related feedback from the immediate environment, i.e. the technical system). Yet we demonstrated deactivation for all sub-regions except area d23, which showed the described positive deflection in the BOLD response. This result suggests to incorporate a further distinction between areas d23 and d31 within the dorsal PCC in functional conceptualisations of the PCC such as the ABBA model.

With the conceived role of area d23 in behavioural adaptation in mind, the question arises why PCC effects such as the ones observed here have not been reported earlier in the feedback processing literature. Depending on the particular study at hand, this gap may be attributed to one or more of several reasons. For example, many reward-related studies did not include any neutral or registering feedback in their experimental setup^24,26,37,39,41,64,65^ or neglected contrasting it to evaluative feedback directly in favour of other comparisons more imperative to the respective publication^23,66^. Others did investigate the neural correlates of neutral versus evaluative feedback but selectively focused on specific volumes of interest like striatal regions, excluding the PCC from analysis^40^. One previous study^44^ reporting whole-brain effects besides the targeted differences in the striatum did in fact list an increased activation of PCC region BA23 in a comparison of evaluative feedback with a ‘no-feedback’ condition (where a pound sign was shown after both correct and incorrect responses). However, since the study’s focus was on the striatal activation differences, the observed involvement of the PCC was not discussed any further. Finally, many previous studies on feedback processing, even if they did implement neutral feedback in a direct comparison with positive and negative feedback (associated with monetary gain and loss), set up experimental contexts where the evaluative feedback was not contingent upon the participants’ actual responses (e.g. in gambling-like tasks like the card-guessing paradigm)^22,38,39,41^. Therefore, this type of feedback did not contain information that could be utilised for monitoring task performance, which may be the reason why these studies did not observe dorsal PCC activation.

In summary, our results do not support the hypothesis that the informational value of (computer) feedback merely constitutes some kind of reward, because then more informative feedback should have led to increased activation of dopaminergic brain regions such as the dorsal striatum. Instead, the current results show that, independent of their informational content, the two types of feedback activate the dorsal striatum to an equal extent which might reflect a more basic process indicating the successful continuation of an interaction. This does not rule out the possibility of additional modulations of striatal activation when feedback is combined with primary or secondary reinforcers or when it signals a step towards attaining a rewarding outcome. The informational value of feedback, on the other hand, appears to be independently encoded in the dorsal portion of the posterior cingulate cortex (area d23), which responds more strongly to the additional information contained in evaluative feedback as compared to registering feedback. This supports the role of this brain region in the processing of information that is potentially relevant for behavioural adjustments.

Considering the importance of the informational value of feedback suggested by the present results, a more comprehensive examination of informative feedback would be conducive to attain a thorough understanding of the complex neuronal differentiation between feedback mechanisms. This includes the direct comparison to monetary gains and losses, the use of uninformative, non-contingent feedback and no feedback as additional control conditions, as well as a differentiation between positive and negative feedback trials by using an event-related fMRI design with larger inter-trial intervals than the ones used in the current block design. Furthermore, examining larger groups of participants and including a manipulation of task difficulty might uncover additional brain regions besides the dPCC that are modulated by the informational value of feedback. Finally, we would like to argue that the interpretation of results on the neural correlates of feedback functions is not necessarily restricted to interactions within computer-controlled settings as used in most neuropsychological studies. The observation that feedback from technical systems is perceived and processed in a remarkably similar way to feedback from human interaction partners (see the CASA—Computers Are Social Actors—paradigm^67,68^) suggests that such results offer insights into more general brain mechanisms of the various functions of feedback.

## Methods

### Participants

Twenty-three right-handed subjects (Edinburgh Handedness Inventory^69^) volunteered to participate in the experiment. Six participants were excluded from further analysis because of excessive head motion^70^ in either of the two fMRI sessions (see below), and one participant due to technical difficulties. Thus, the data of 16 participants (eight females and eight males, aged 21–30 years, mean age $26.15 \pm 2.80$ years) entered analysis. All participants gave written informed consent to the study, which was approved by the ethics committee of the University of Magdeburg, Germany, and conducted in accordance with the regulations of the Declaration of Helsinki.

### Stimuli and task

Upward and downward frequency modulated (FM) tones served as auditory stimuli. These FM tones had a duration of 400 ms and differed in centre frequency ($\hbox {F}_{\rm {c}} = 500-3800\,\hbox {Hz}$ in steps of 100 Hz). The starting and end frequencies of the tones were calculated by $\hbox {F}_{\rm {c}}\,\hbox {[Hz]}\,\pm \,\hbox {F}_{\rm {c}}\,\hbox {[Hz]}\,\times \,\hbox {k}^{-1}\,\times \,\hbox {duration [s]}$, with k = 2 or k = 4. Participants were required to categorise the FM tones according to the direction of modulation (upward vs. downward). They had to press a button with the right index finger in response to upward modulated FM tones and another button with their right middle finger indicating downward modulated FM tones. Immediately following the button press, system feedback was presented. Feedback in the blocks of stimuli differed with regard to informational content and modality as specified below.

The experiment was divided into two functional runs that differed in the feedback’s informational content. One half of all participants first received registering feedback and then evaluative feedback. The other half of the participants received evaluative feedback first. Registering feedback only indicated that the system has registered the participant’s response without evaluating the correctness of their input. Evaluative feedback indicated if their input was correct or incorrect. Within each of the two consecutive runs, 24 blocks of stimuli were presented. Each block consisted of 16 FM tones presented with an inter-stimulus interval of 2 s. The blocks differed in feedback modality (eight blocks in each modality) and were presented in a pseudo-randomised order, alternating with resting blocks of 20 s. Depending on the block, feedback was presented either verbally (spoken feedback), in non-verbal auditory form (pure tones) or visually (symbols). Table 2 summarises the different types of feedback. Visual and auditory feedback were presented for 0.5 s. Verbal feedback had a similar duration ($569\,\hbox {ms}~\pm ~26\,\hbox {ms}$).

**Table 2:** Feedback types by modality and informational content.

Modality	Registering feedback	Evaluative feedback	
		Positive	Negative
Visual			
Auditory	Pure tone, 330 Hz	Pure tone, 440 Hz	Pure tone, 220 Hz
Verbal	“Okay”	“Yes”	“No”

During the entire experiment (with the exception of the periods of feedback presentation in the visual modality blocks), participants were asked to look at a white fixation cross on grey background.

### Data acquisition

The measurements were carried out in a 3 Tesla scanner (MAGNETOM Trio, Siemens Healthcare GmbH, Erlangen, Germany) equipped with an eight channel head coil. A 3D anatomical data set of the participant’s brain (echo time (TE), 4.77 ms; repetition time (TR), 2500 ms; flip angle, $7^{\circ }$; matrix size, $256\times 256$ voxels; field of view, $25.6\times 25.6\,\hbox {cm}^{2}$; 192 slices of 1 mm thickness) was obtained before the functional measurement. Additionally, a 2D anatomical data set was acquired before each of the two functional measurements using inversion-recovery echo-planar imaging (IR-EPI). The IR-EPI has the same geometric distortions as the functional measurement but a reversed contrast and thus allows a more precise coregistration of the functional data to the anatomical data. The two functional measurements each consisted of 634 functional volumes that were acquired in 21 min 8 s using an echo planar imaging (EPI) sequence (TE, 30 ms; TR, 2000 ms; flip angle, $80^{\circ }$; 3 mm isotropic resolution; 32 slices; 0.3 mm gaps).

The head of the participant was fixed with a cushion with attached ear muffs containing fMRI compatible headphones^71^. Additionally, the participants wore earplugs that, together with the headphones, reduced the scanner noise by 40–60 dB. The software Presentation (Neurobehavioral Systems Inc., Berkeley, CA) was used for stimulus presentation and the recording of behavioural responses. Before the experiment, the overall stimulus intensity was adjusted to a comfortable level for each participant and equally loud at both ears. Visual stimuli were presented by a video projector onto a back projection screen, which was visible inside the scanner via a mirror system.

### Data analysis

#### Behavioural data

Mean reaction times and error rates of each participant for each experimental condition were calculated from the behavioural log data of the fMRI experiments. These behavioural data were then further analysed with SPSS (IBM Corp., Armonk, NY) and examined for normal distribution by visual inspection of Q–Q plots, Shapiro-Wilk tests and evaluation of skewness and kurtosis. $2\times 3$ factorial repeated-measures analyses of variances (ANOVAs) were conducted to examine significant differences in accuracy and reaction times depending on informational content (registering vs. evaluative feedback) and modality (visual vs. auditory vs. verbal feedback). To counteract violations of sphericity in effects including the 3-level factor modality, Greenhouse–Geisser (G–G) corrections were applied.

#### fMRI data

The functional data were analysed with the software BrainVoyager QX (Brain Innovation B.V., Maastricht, The Netherlands). A standard sequence of preprocessing steps, i.e. slice scan time correction, 3D-motion correction, linear trend removal, spatial smoothing with a Gaussian filter of 4 mm full width at half maximum, and temporal filtering with a high-pass of three cycles per scan was performed^10^. The functional data were inspected thoroughly for severe grey level fluctuations resulting from head motion. For that purpose, the automated head motion correction procedure, which resulted in estimated translation and rotation parameters for each spatial direction, was analysed^72^. Subjects with head movements that exceeded $2^{\circ }$ rotation or 3.0 mm translation in any direction in either of the two functional runs (evaluative feedback, registering feedback) were excluded from further analysis^70^. Finally, the functional data were co-registered with the 3D anatomical data by utilising the IR-EPI, and then transformed into Talairach-space^73^.

A random-effects analysis with a general linear model (GLM) including both *z*-transformed functional data sets of the 16 participants, was performed for all task conditions using the 2-gamma response function implemented in BrainVoyager QX. The model included one predictor for each of the six experimental conditions. To reduce signal artefacts from brain areas with low signal intensity, only those voxels were considered whose functional EPI signal had a grey level of at least 75.

To investigate the differential effects of evaluative versus registering feedback across modalities, the three modality-specific contrasts between evaluative feedback and registering feedback, and the three modality-specific contrasts between evaluative feedback and rest were combined in a conjunction analysis. To correct for multiple comparisons, a false discovery rate (FDR) of $\textit{q} = 0.01\ (\textit{t} > 3.40, \textit{p} <0 .004)$ was applied. Volumes-of-interest (VOIs) were defined as all resulting clusters that comprised at least $54\,\hbox {mm}^{3}$.

## Data Availability

The data that support the findings of this study are available from the corresponding author upon reasonable request.
